# Changes in the Use of Montelukast for Asthma After a US Food and Drug Administration Boxed Warning

**DOI:** 10.1001/jamanetworkopen.2026.14274

**Published:** 2026-05-22

**Authors:** Hariharan Shanmugam, Aaron S. Kesselheim, Ian T. T. Liu, William B. Feldman, Benjamin N. Rome

**Affiliations:** 1 Program On Regulation, Therapeutics, And Law (PORTAL), Division of Pharmacoepidemiology and Pharmacoeconomics, Department of Medicine, Brigham and Women’s Hospital and Harvard Medical School, Boston, Massachusetts; 2 Division of Pulmonary, Critical Care, Sleep Medicine, Clinical Immunology, and Allergy, Department of Medicine, University of California, Los Angeles

## Abstract

**Importance:**

In March 2020, the US Food and Drug Administration (FDA) issued a boxed warning for montelukast amid reports of neuropsychiatric adverse effects.

**Objective:**

To determine whether a boxed warning for montelukast was associated with changes in prescription rates of montelukast to treat asthma in the US.

**Design, Setting, and Participants:**

In this serial cross-sectional study using a national commercial insurance claims database, monthly cohorts of individuals aged 6 years and older with an asthma diagnosis and continuous insurance coverage in the past year were created from October 2017 to December 2022. Results were stratified by age group, inhaled corticosteroid (ICS) use, and 1 or more asthma-related emergency department visit or hospitalization in the prior year. Data were analyzed from August 2024 to June 2025.

**Exposures:**

Announcement of a boxed warning for montelukast in March 2020.

**Main Outcomes and Measures:**

Monthly incidence and prevalence of montelukast use among patients with asthma. Data were analyzed with an interrupted time series analysis using segmented ordinary least-squares linear regression models with Newey-West standard errors.

**Results:**

The number of patients with asthma ranged from 594 253 in October 2017 to 614 637 in December 2022. In October 2017, 348 359 patients (58.6%) were female, 113 913 (19.2%) were younger than age of 18 years, 292 730 (49.3%) used an ICS, and 105 869 (17.8%) had 1 or more asthma-related emergency department visit or hospitalization in the prior year. The baseline monthly prevalence of montelukast use was 160.0 per 1000 patients with asthma, and the baseline incidence of new montelukast use was 5.8 per 1000. After the boxed warning, the level of monthly incident montelukast prescriptions decreased by 1.7 (95% CI, −2.2 to −1.3) per 1000 and the trend in montelukast prevalence decreased by 0.9 (95% CI, −1.1 to −0.6) per 1000 per month relative to the prewarning trend. The trend in prevalent montelukast use decreased after the boxed warning across all age and asthma severity subgroups.

**Conclusions and relevance:**

In this cross-sectional study of commercially insured patients with asthma, an FDA boxed warning for montelukast was associated with changes in asthma treatment use among patients of all ages.

## Key Points

**Question:**

Did the use of montelukast to treat asthma change after an US Food and Drug Administration (FDA) boxed warning was announced in March 2020?

**Findings:**

In this cross-sectional study using national monthly cohorts of up to 614 637 patients with asthma from a national commercial claims dataset, the use of montelukast decreased after implementation of an FDA boxed warning.

**Meaning:**

These findings suggest that treatment patterns for patients with asthma changed after an FDA boxed warning.

## Teaser

This cross-sectional study examines whether a US Food and Drug Administration (FDA) boxed warning for montelukast was associated with changes in prescription rates of montelukast to treat asthma in the US.

## Introduction

Montelukast is a leukotriene receptor antagonist used primarily to treat asthma, exercise-induced bronchospasm, and allergic rhinitis. After its approval by the US Food and Drug Administration (FDA) in 1998, reports of potential neuropsychiatric adverse effects to montelukast emerged, including depression, aggressive behavior, and suicidal ideation.^1^ In 2008 and 2009, the FDA issued safety alerts about certain potential neuropsychiatric adverse effects and began collecting data about these adverse events through its Sentinel monitoring initiative.^2^ To date, published evidence about whether montelukast causes neuropsychiatric adverse effects has been mixed, with several studies and a systematic review showing no association^1,3,4^ and other studies showing increased risk.^5,6,7^

In March 2020, the FDA announced the addition of a boxed warning (colloquially known as a black box warning) to the labeling of montelukast about potential neuropsychiatric adverse effects, advising that its use in asthma be determined on a case-by-case basis and that use in allergic rhinitis be reserved only for those who did not experience improvement with other therapies; this warning was formally added to the labeling of montelukast in April 2020.^8^ The boxed warning is the FDA’s highest level of caution, used to highlight serious adverse effects that patients should know if they were considering taking the drug.

Prior studies of boxed warnings have suggested a mixed impact on prescription rates. For example, some studies examining the addition of boxed warnings to the labeling of selective serotonin reuptake inhibitors^9^ and dopamine antagonist promotility agents^10^ were followed by lower reduced prescription rates. By contrast, boxed warnings did not measurably affect prescription rates of bupropion^11^ or periprocedural tramadol.^12^ Even within a single class of drugs, the impact of boxed warnings can vary; warnings about fluoroquinolone antibiotics associated with neurologic adverse effects were associated with lower prescribing rates,^13^ but a later-added warning about aortic dissections was not.^14^

The impact of a boxed warning on the use of montelukast has not been studied but may have important effects on the management of asthma and allergic rhinitis. Therefore, we aimed to measure changes in montelukast use for asthma after implementation of the FDA boxed warning.

## Methods

### Study Design and Cohort

We performed a retrospective serial cross-sectional study using US national commercial claims data from Merative MarketScan Research Databases from October 2017 to December 2022 (most recent available data at the time of analysis). We created monthly cohorts of individuals older than age 6 years with an *International Statistical Classification of Diseases and Related Health Problems, Tenth Revision* (*ICD-10*) diagnosis code for asthma and use of 1 or more inhaled medication used to treat asthma within the prior 365 days. We excluded individuals who did not have continuous insurance coverage throughout the month of interest and the preceding 365 days, which allowed us to measure covariates and assess whether patients were new or prevalent montelukast users. Individuals could be included in multiple monthly cohorts. The study was deemed exempt from review and informed consent by the Mass General Brigham institutional review board because it used deidentified data. This cross-sectional study followed the Strengthening the Reporting of Observational Studies in Epidemiology (STROBE) reporting guideline.

### Primary Outcomes

The primary outcomes were incidence and prevalence of montelukast use among those with asthma. Prevalent use was defined as the proportion of patients with asthma with an available filled prescription for montelukast during each calendar month, including prescriptions that were filled earlier if the days’ supply extended into the calendar month. Incident use was defined as the number of patients who filled a new prescription for montelukast in the calendar month, among the subset of those with asthma who had had not filled a prescription for montelukast or another leukotriene receptor antagonist (zafirlukast or zileuton) during the prior 365 days.

### Exposure and Covariates

The exposure was the FDA announcement of the new boxed warning in March 2020, with the postexposure period beginning in April 2020. To assess for potential confounding due to a changing asthma population over time, we measured monthly changes in patient covariates, including age, sex, geographic US census region, and use of other asthma medications. These other asthma medications included inhaled short-acting beta agonists, long-acting beta agonist, short-acting muscarinic antagonists, long-acting muscarinic antagonists, and corticosteroids; combination inhalers were counted toward each component drug class. We also included other noninhaled asthma therapies, including theophylline, alternative leukotriene receptor antagonists (zafirlukast and zileuton), and biologics, including benralizumab, dupilumab, mepolizumab, omalizumab, reslizumab, and tezepelumab-ekko. We compared differences between characteristics of the cohorts in October 2017 and October 2022 using standardized mean differences.

Including time-varying potential confounders in time series models is problematic if they are mediators, so instead we conducted stratified analyses by age group (younger than 18 years, 18 to 64 years, and 65 years or older) and by markers of asthma severity. These markers included whether patients had an asthma-related emergency department (ED) visit or hospitalization (defined as encounters including an *ICD-10* code for asthma or shortness of breath) and whether patients filled a prescription for inhaled corticosteroids in the prior 365 days. These measures were chosen because they are similar to criteria used to stratify asthma severity in the National Asthma Education and Prevention Program guidelines that outlined the standard-of-care treatment during this study period.^15^

### Statistical Analysis

We performed an interrupted time series analysis using segmented ordinary least-squares linear regression models with Newey-West standard errors to measure changes in the monthly incidence and prevalence of montelukast use after the implementation of the boxed warning in March 2020. Models were adjusted for seasonality and first-order autocorrelation, as determined by the Cumby-Huizinga test.

To assess for potential temporal confounding from factors that occurred simultaneous to the boxed warning (eg, the onset of policies associated with the COVID-19 pandemic), we performed a sensitivity analysis in which we measured changes in monthly montelukast use compared with changes in monthly use of other noninhaled asthma medications that were not subject to the March 2020 boxed warning. These other medications included theophylline, zafirlukast, zileuton, benralizumab, dupilumab, mepolizumab, omalizumab, reslizumab, and tezepelumab-ekko. The FDA Drug Safety-Related Labeling Changes database was checked to ensure that none of these other asthma drugs had a major safety labeling change during the study period. In a separate analysis, we excluded zafirlukast and zileuton from the comparator group because use of these medications with similar mechanisms to montelukast may have been affected by the boxed warning. We conducted a separate sensitivity analysis including a 2-month washout of March and April 2020 (ie, May 2020 served the first month of the postintervention period), to account for the 1-month lag between the boxed warning announcement in March 2020 and physical labeling change in April 2020. Analyses were conducted using StataSE version 16 (StataCorp). Statistical significance was set at *P* < .05, and all tests were 2-sided. Data were analyzed from August 2024 to June 2025.

## Results

### Cohort Characteristics

The number of patients with asthma included in each monthly cohort ranged from 594 253 in October 2017 to 613 152 in October 2022. In October 2017, 348 359 (58.6%) of individuals in the overall asthma cohort were female, 113 913 (19.2%) were less than 18 years old, 292 730 (49.3%) used an ICS in the prior year, and 105 869 (17.8%) had 1 or more recent asthma-related ED visit or hospitalization (Table 1). Overall, characteristics of the cohort were similar over the study period; nearly all standardized mean differences between October 2017 and October 2022 cohorts were less than 0.1.

**Table 1.** Characteristics of Patients With Asthma, Selected Monthly Cohorts, 2017-2022

Characteristic	Patient, No. (%)						SMD, October 2017 vs October 2022^a^
	October 2017 (n = 594 253)	October 2018 (n = 704 816)	October 2019 (n = 723 448)	October 2020 (n = 747 829)	October 2021 (n = 644 553)	October 2022 (n = 613 152)	
Age at cohort entry, y							
<18	113 913 (19.2)	137 820 (19.6)	140 989 (19.5)	113 500 (15.2)	87 049 (13.5)	92 617 (15.1)	0.108
18-64	390 000 (65.6)	494 400 (70.1)	505 983 (69.9)	524 338 (70.1)	464 342 (72.0)	428 364 (69.9)	0.091
≥65	90 340 (15.2)	72 596 (10.3)	76 476 (10.6)	109 991 (14.7)	93 162 (14.5)	92 171 (15.0)	0.005
Sex							
Male	245 894 (41.4)	290 408 (41.2)	297 431 (41.1)	299 062 (40.0)	261 341 (40.5)	244 383 (39.9)	0.031
Female	348 359 (58.6)	414 408 (58.8)	426 017 (58.9)	448 767 (60.0)	383 212 (59.5)	368 769 (60.1)	
Geographic region							
Northeast	77 480 (13.0)	136 633 (19.4)	134 021 (18.5)	134 502 (18.0)	85 046 (13.2)	89 059 (14.5)	0.043
Midwest	123 425 (20.8)	154 759 (22.0)	152 357 (21.1)	167 865 (22.4)	116 349 (18.1)	99 357 (16.2)	0.118
South	191 531 (32.2)	217 716 (30.9)	228 727 (31.6)	235 126 (31.4)	240 294 (37.3)	204 945 (33.4)	0.025
West	83 985 (14.1)	103 521 (14.7)	101 827 (14.1)	97 354 (13.0)	81 169 (12.6)	77 101 (12.6)	0.046
Unknown	117 832 (19.8)	92 187 (13.1)	106 516 (14.7)	112 982 (15.1)	121 695 (18.9)	142 690 (23.3)	0.084
≥1 Asthma-related ED visit or hospitalization	105 869 (17.8)	129 241 (18.3)	129 230 (17.9)	145 912 (19.5)	149 827 (23.2)	126 588 (20.6)	0.072
Use of asthma medications							
SABA	477 821 (80.4)	593 300 (84.2)	606 089 (83.8)	627 135 (83.9)	533 101 (82.7)	504 151 (82.2)	0.047
LABA	189 856 (31.9)	216 843 (30.8)	230 733 (31.9)	256 061 (34.2)	222 295 (34.5)	205 667 (33.5)	0.034
ICS	292 730 (49.3)	337 451 (47.9)	350 629 (48.5)	370 527 (49.5)	313 784 (48.7)	293 577 (47.9)	0.028
LAMA	39 864 (6.7)	39 791 (5.6)	42 707 (5.9)	52 725 (7.1)	51 029 (7.9)	49 696 (8.1)	0.053
SAMA	45 339 (7.6)	51 202 (7.3)	53 539 (7.4)	56 334 (7.5)	44 481 (6.9)	45 040 (7.3)	0.011
Biologic	3357 (0.6)	4585 (0.7)	6210 (0.9)	8402 (1.1)	8953 (1.4)	9910 (1.6)	0.101
Theophylline	2592 (0.4)	1986 (0.3)	1786 (0.2)	1877 (0.3)	1411 (0.2)	1175 (0.2)	0.044
Zafirlukast	995 (0.2)	836 (0.1)	802 (0.1)	993 (0.1)	673 (0.1)	493 (0.1)	0.025
Zileuton	223 (0.04)	205 (0.03)	176 (0.02)	149 (0.02)	74 (0.01)	77 (0.01)	0.016

Abbreviations: ED, emergency department; ICS, inhaled corticosteroids; LABA, long-acting beta agonist; LAMA, long-acting muscarinic antagonist; SABA, short-acting beta agonist; SAMA, short-acting muscarinic antagonist; SMD, standardized mean difference.

^a^ An SMD of <0.1 denotes negligible imbalance between groups.

Among montelukast users, characteristics were generally similar throughout the study period, although there were fewer pediatric users in more recent months (18 802 patients [17.8%] in October 2017 vs 10 583 [11.1%] in October 2022) (eTable 1 in Supplement 1). Compared with the overall cohort of patients with asthma, prevalent montelukast users were less likely to have had an ED visit or hospitalization for asthma in the past year (eg, 16 977 [16.1%] vs 105 869 [17.5%] in October 2017) and more likely to be using an ICS (eg, 71 974 [68.2%] vs 292 730 [49.3%] in October 2017).

### Interrupted Time Series Analysis

**Figure 1.**
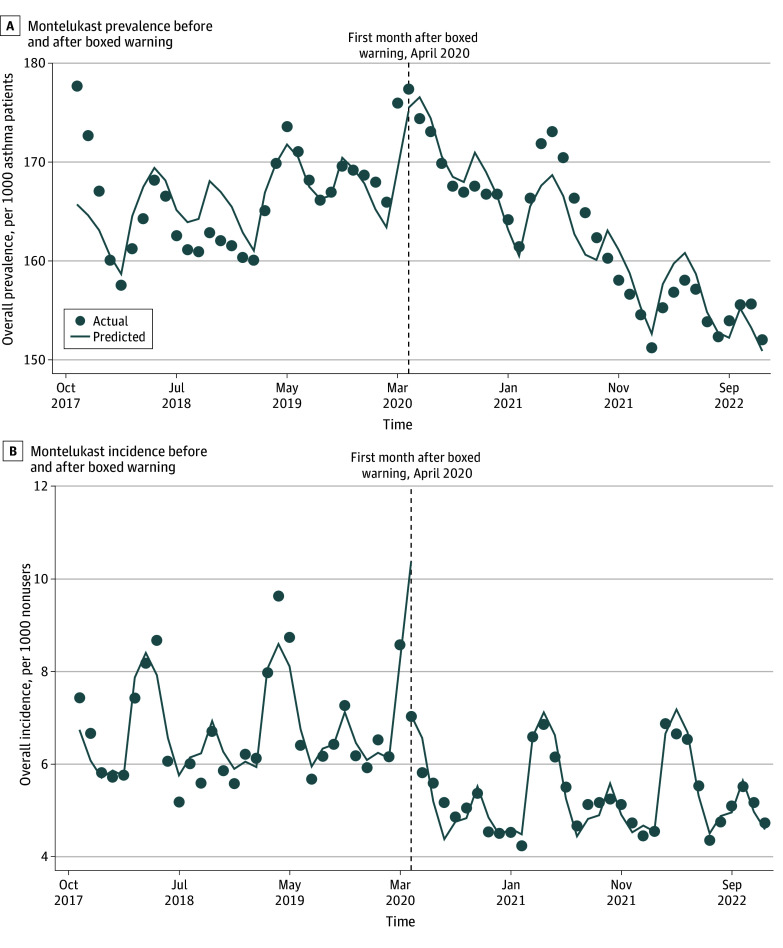
Line Graph of Montelukast Use Before and After March 2020 Boxed Warning Dots represent actual values from the monthly cohorts and lines represent estimated values from linear models that were adjusted for seasonality and first-order autocorrelation.

Figure 1 shows trends in the monthly prevalence and incidence of montelukast use among patients with asthma over the study period. The modeled baseline monthly prevalence of montelukast use was 160.0 (95% CI, 155.1 to 164.8) per 1000 patients, and the modeled baseline monthly incidence was 5.8 new users per 1000 (Table 2). There were no significant nonzero trends in prevalence or incidence in the baseline period. After the boxed warning, there was no change in the level of the monthly prevalence of montelukast but the monthly trend decreased by 0.9 (95% CI, −1.1 to −0.6) per 1000; in the last month of the study (December 2022), the estimated prevalence was 151.7 (95% CI, 148.7 to 154.5) per 1000. The level of monthly montelukast incidence decreased by 1.7 (95% CI, −2.2 to −1.3) per 1000 patients after the boxed warning, with no significant changes in trend.

**Table 2.** Interrupted Time Series Analysis Estimates of Changes in Montelukast Use After March 2020 Boxed Warning Announcement^a^

Characteristic	Baseline level, per 1000 patients per month (95% CI)	Baseline trend, per 1000 patients per month (95% CI)	*P* value	Postwarning change in level, per 1000 patients (95% CI)^b^	*P* value	Postwarning change in trend, per 1000 patients per month (95% CI)^c^	*P* value	Postwarning trend, per 1000 patients per month (95% CI)^d^	*P* value
All patients									
Montelukast prevalence	160.0 (155.1 to 164.8)	0.2 (−0.1 to 0.5)	.18	3.3 (−0.8 to 7.3)	.12	−0.9 (−1.1 to −0.6)	<.001	−0.7 (−0.8 to −0.6)	<.001
Montelukast incidence (per 1000 nonusers per month)	5.8 (5.4 to 6.2)	0.16 (−0.004 to 0.04)	.13	−1.7 (−2.2 to −1.3)	<.001	−0.01 (−0.03 to 0.01)	.37	−0.005 (−0.009 to 0.02)	.48
Montelukast prevalence, by subgroups									
Age, y									
<18	142.4 (136.9 to 148.0)	−0.2 (−0.6 to 0.05)	.11	−5.7 (−11.9 to 0.5)	.07	−0.5 (−0.9 to −0.1)	.006	−0.8 (−1.0 to −0.5)	<.001
18-64	163.1 (157.6 to 168.6)	0.3 (0.07 to 0.7)	.045	2.3 (−2.1 to 6.7)	.30	−1.0 (−1.3 to −0.7)	<.001	−0.7 (−0.8 to −0.5)	<.001
≥65	172.1 (170.0 to 174.3)	0.04 (−0.06 to 0.1)	.45	7.8 (4.4 to 11.1)	<.001	−0.7 (−0.9 to −0.5)	<.001	−0.7 (−0.8 to −0.5)	<.001
≥1 asthma-related ED visit or hospitalization									
No	162.8 (157.3 to 168.3)	0.2 (−0.1 to 0.5)	.24	6.5 (1.4 to 11.7)	.01	−0.8 (−1.1 to −0.4)	<.001	−0.6 (−0.7 to −0.4)	<.001
Yes	147.7 (143.8 to 151.5)	0.2 (0.04 to 0.4)	.01	−7.9 (−13.0 to −2.8)	.002	−1.1 (−1.5 to −0.7)	<.001	−0.9 (−1.2 to −0.6)	<.001
ICS use									
No	97.0 (93.2 to 100.8)	0.08 (−0.1 to 0.3)	.48	−0.2 (−3.3 to 2.9)	.89	−0.3 (−0.5 to −0.02)	.03	−0.2 (−0.3 to −0.1)	.001
Yes	228.8 (224.0 to 233.6)	0.3 (−0.007 to 0.6)	.06	3.1 (−1.1 to 7.2)	.15	−1.3 (−1.6 to −1.0)	<.001	−1.0 (−1.1 to −0.9)	<.001

Abbreviations: ED, emergency department; ICS, inhaled corticosteroids.

^a^ All analyses adjusted for seasonality and first-order autocorrelation.

^b^ Estimates of the postintervention linear trend were obtained using linear combinations of the preintervention trend and the change in trend.

^c^ Trend changes reflect the estimated change in the slope of prescription rates per 1000 between the prewarning period (October 2017 to March 2020, inclusive) and the postwarning period (April 2020 to December 2022).

^d^ Estimates of the postintervention linear trend were obtained using linear combinations of the preintervention trend and the change in trend.

**Figure 2.**
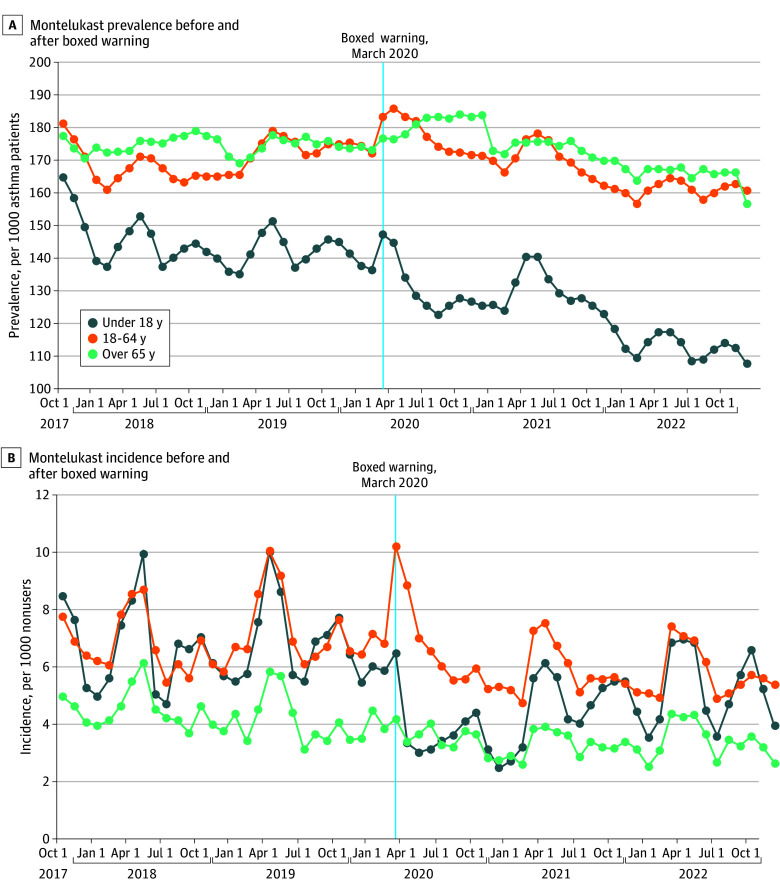
Line Graph of Montelukast Use Before and After 2020 Boxed Warning by Age Group Estimated values are from linear models that were adjusted for seasonality and first-order autocorrelation.

The downward change in the trend in monthly montelukast prevalence was observed in all age groups and when stratifying patients by markers of asthma severity (Table 2). There was a more abrupt decline in use of montelukast among those aged younger than 18 years (Figure 2). There were variations in the level changes between subgroups, although all level changes were small (less than 5% of the baseline prevalence).

### Sensitivity Analyses

The monthly prevalence of montelukast was substantially higher than other noninhaled asthma medications in the baseline period, although the use of other noninhaled medications was increasing (eFigure 1 and eTable 2 in Supplement 1). After the announcement of the montelukast boxed warning, there was a slightly positive change in the monthly trend of the prevalence of noninhaled asthma medications (0.1 [95% CI, 0.004 to 0.1] per 1000; *P* = .03); the trend in monthly montelukast prevalence decreased by 0.9 (95% CI, −1.2 to −0.6) per 1000, relative to the change in trend for other noninhaled medications. These results were similar in sensitivity analyses that excluded zileuton and zafirlukast from the comparator group (eTable 3 in Supplement 1) and in a separate sensitivity analysis that included a 2-month washout period (eTable 4 in Supplement 1).

## Discussion

This interrupted time series analysis found that an FDA boxed warning was associated with reductions in montelukast use among commercially insured patients with asthma. These results suggest that the FDA’s boxed warning influenced prescribing patterns, even though causal evidence linking montelukast with neuropsychiatric adverse effects is lacking.

These findings were consistent with past studies for other drugs that have occasionally found a meaningful impact of FDA boxed warnings on medication use. For example, boxed warnings were associated with modest decreases in prescription rates for fluoroquinolones^13^ and a substantial decrease in prescription rates for metoclopramide for gastroparesis.^10^ Whether a boxed warning affects prescribing likely varies based on drug-specific contextual factors, including the severity of the potential adverse effect, the quality of evidence, the availability of alternatives, and how well the warning was communicated to prescribers.^16^ Further quantitative studies of these cases and qualitative surveys of the prescribers of these medications can help pinpoint the relevant factors and help the FDA plan for future cases in which widespread warning about emerging safety issues is needed.

In the case of montelukast, incident use decreased by nearly 30% in the immediate aftermath of the FDA warning and remained at this lower level for 2 years. Two years after the warning, prevalent use of the drug declined by about 5%, suggesting that many patients with asthma who were taking montelukast at the time of the warning continued to do so after the warning. This may be because patients and clinicians were unaware of the warning,^17^ or felt the benefits continued to outweigh the risks if no neuropsychiatric adverse effects were being experienced. Additionally, montelukast is frequently prescribed for patients who did not experience improvement with prior therapeutic options, so the relatively small overall reduction in prevalent use of montelukast may reflect limited treatment options for refractory asthma. With the emergence of new biologic treatments that provide additional alternatives for refractory patients with asthma,^18^ use of montelukast may continue to decline further in future years.

The decrease in montelukast use around the time of the boxed warning was observed across all age groups. The decline was more abrupt among those younger than 18 years, perhaps because there was particular attention in the lay media on the experience of neuropsychiatric adverse effects in children.^17^ However, the declining use was observed across different age groups and severity levels; this suggests it may be difficult for the FDA to tailor these warnings to specific subgroups of patients when needed. We observed a small but statistically significant transient increase in the level of montelukast prevalence among adults aged 65 years or older immediately following the boxed warning. Since the boxed warning coincided with the onset of the COVID-19 pandemic in March 2020, this finding may be associated with short-term changes in prescription dynamics due to new interest in the use of montelukast for COVID-19^19^ and further exacerbated by the smaller size of the 65 years or older subgroup relative to the other age subgroups. Notably, the postwarning trend in this group remained negative, consistent with the overall findings.

Particularly in the setting of mixed clinical evidence, boxed warnings should be as tailored as possible to the groups for which data are most compelling. In the case of montelukast, since supporting data are mixed, further research into the association between the drug and neuropsychiatric adverse effects is warranted. While adding a boxed warning remains a reasonable precautionary step given the potential severity of this adverse effect, it is important to continue pharmacovigilance efforts, which may lead to adjustments in the wording or even removal of the boxed warning in the future.

### Limitations

This study has limitations. The data were from commercially insured patients and may not be generalizable to those with public insurance (eg, Medicaid) or uninsured patients. Additionally, our case definition of asthma may be overly inclusive. There were small changes in the population of patients with asthma over the study period, although results were consistent across stratified analyses. Time series analyses cannot fully isolate the effects of the intervention from other co-occurring events. In this case, the FDA boxed warning coincided with the onset of the COVID-19 pandemic, which had profound impacts on access to care in 2020. However, a sensitivity analysis showed that the use of other noninhaled asthma medications continued to increase after March 2020, suggesting the results for montelukast were likely associated with the boxed warning.

## Conclusions

In this serial cross-sectional study of commercially insured patients with asthma, the use of montelukast decreased after the implementation of an FDA boxed warning. Boxed warnings can have meaningful impacts on prescribing practices and thus should be carefully tailored to available evidence. Lessons from the case of montelukast should be applied to future scenarios that call for regulatory action.

## Notes

Supplement 1. **eTable 1.** Characteristics of selected monthly cohorts of prevalent Montelukast users, 2017-2022 **eFigure.** Trends in prevalence of montelukast and noninhaled asthma medication controls before and after March 2020 boxed warning **eTable 2.** Multiple-group ITSA of changes in Montelukast prevalence after boxed warning relative to controls **eTable 3.** Multiple-group ITSA of changes in Montelukast prevalence after boxed warning relative to controls, excluding zafirlukast and zileuton **eTable 4.** Interrupted time series analysis estimates of changes in Montelukast use after March 2020 boxed warning announcement, with washout period

Supplement 2.
Data Sharing Statement
