# Supplementary material for: Changes in the Use of Montelukast for Asthma After a US Food and Drug Administration Boxed Warning
Source: JAMA Netw Open. 2026 May 22;9(5):e2614274. doi: 10.1001/jamanetworkopen.2026.14274 (PMC13197876; doi:10.1001/jamanetworkopen.2026.14274)
Supplement: Supplement 1. — eTable 1. Characteristics of selected monthly cohorts of prevalent Montelukast users, 2017-2022 eFigure. Trends in prevalence of montelukast and noninhaled asthma medication controls before and after March 2020 boxed warning eTable 2. Multiple-group ITSA of changes in Montelukast prevalence after boxed warning relative to controls eTable 3. Multiple-group ITSA of changes in Montelukast prevalence after boxed warning relative to controls, excluding zafirlukast and zileuton eTable 4. Interrupted time series analysis estimates of changes in Montelukast use after March 2020 boxed warning announcement, with washout period [file jamanetwopen-e2614274-s001.pdf]

Supplemental Online Content

Shanmugam H, Kesselheim AS, Liu ITT, Feldman WB, Rome BN. Changes in the use of montelukast for asthma after a US Food and Drug Administration boxed warning. *JAMA Netw Open*. 2026;9(5):e2614274. doi:10.1001/jamanetworkopen.2026.14274

eTable 1. Characteristics of selected monthly cohorts of prevalent Montelukast users, 2017-2022

eFigure. Trends in prevalence of montelukast and noninhaled asthma medication controls before and after March 2020 boxed warning

eTable 2. Multiple-group ITSA of changes in Montelukast prevalence after boxed warning relative to controls

eTable 3. Multiple-group ITSA of changes in Montelukast prevalence after boxed warning relative to controls, excluding zafirlukast and zileuton

eTable 4. Interrupted time series analysis estimates of changes in Montelukast use after March 2020 boxed warning announcement, with washout period

This supplemental material has been provided by the authors to give readers additional information about their work.

Supplemental Table 1. Characteristics of selected monthly cohorts of prevalent Montelukast users, 2017 - 2022

	October 2017	October 2018	October 2019	October 2020	October 2021	October 2022
**N (%)**	105,601	114,847	122,667	125,299	103,307	95,426
**Age at cohort entry**						
Under 18 years	18,802 (17.8%)	19,963 (17.4%)	20,580 (16.8%)	14,528 (11.6%)	10,946 (10.6%)	10,583 (11.1%)
18-64 years	70,741 (67.0%)	81,882 (71.3%)	88,609 (72.2%)	90,514 (72.2%)	76,423 (74.0%)	69,506 (72.8%)
65 years and over	16,058 (15.2%)	13,002 (11.3%)	13,478 (11.0%)	20,257 (16.2%)	15,938 (15.4%)	15,337 (16.1%)
**Female Sex**	67,399 (63.8%)	73,418 (63.9%)	78,611 (64.1%)	82,512 (65.9%)	68,149 (66.0%)	63,204 (66.2%)
**Geographic Region**						
Northeast	11,630 (11.0%)	19,043 (16.6%)	19,146 (15.6%)	19,805 (15.8%)	12,334 (11.9%)	12,310 (12.9%)
Midwest	22,409 (21.2%)	26,570 (23.1%)	26,518 (21.6%)	27,943 (22.3%)	19,766 (19.1%)	16,247 (17.0%)
South	37,037 (35.1%)	39,115 (34.1%)	43,268 (35.3%)	42,036 (33.5%)	39,795 (38.5%)	33,334 (34.9%)
West	11,671 (11.1%)	12,995 (11.3%)	13,597 (11.1%)	12,828 (10.2%)	10,947 (10.6%)	9,999 (10.5%)
Unknown	22,854 (21.6%)	17,124 (14.9%)	20,138 (16.4%)	22,687 (18.1%)	20,465 (19.8%)	23,536 (24.7%)
**Hospitalization for asthma**	16,977 (16.1%)	19,696 (17.1%)	20,520 (16.7%)	21,448 (17.1%)	19,219 (18.6%)	16,908 (17.7%)
**Use of asthma medications**						
SABA	79,151 (75.0%)	91,944 (80.1%)	97,978 (79.9%)	100,890 (80.5%)	81,126 (78.5%)	75,025 (78.6%)
LABA	50,619 (47.9%)	56,037 (48.8%)	61,060 (49.8%)	66,592 (53.1%)	54,654 (52.9%)	48,598 (50.9%)
ICS	71,974 (68.2%)	78,703 (68.5%)	83,933 (68.4%)	87,305 (69.7%)	70,963 (68.7%)	63,117 (66.1%)
LAMA	9,033 (8.6%)	9,571 (8.3%)	10,658 (8.7%)	13,002 (10.4%)	11,962 (11.6%)	11,608 (12.2%)
SAMA	10,234 (9.7%)	11,703 (10.2%)	12,709 (10.4%)	13,388 (10.7%)	10,147 (9.8%)	9,783 (10.3%)
Biologic	1,545 (1.5%)	2,004 (1.7%)	2,581 (2.1%)	3,448 (2.8%)	3,399 (3.3%)	3,684 (3.9%)
Theophylline	1,041 (1.0%)	807 (0.7%)	698 (0.6%)	730 (0.6%)	509 (0.5%)	475 (0.5%)

Abbreviations: SABA = short-acting beta agonist, LABA = long-acting beta agonist, ICS = inhaled corticosteroids, LAMA = long-acting muscarinic antagonist, SAMA = short-acting muscarinic antagonist

© 2026 Shanmugam H et al. *JAMA Network Open*.

Supplemental Figure 1. Trends in prevalence of montelukast and non-inhaled asthma medication controls before and after March 2020 boxed warning

Predicted values are from linear models adjusted for seasonality and first-order autocorrelation.

© 2026 Shanmugam H et al. *JAMA Network Open*.

Supplemental Table 2. Multiple-group ITSA of changes in Montelukast prevalence after boxed warning relative to controls [a]

	**Model estimate, per 1000 asthma patients per month (95% CI)**	***P* value**
Pre-warning baseline level	8.2 (7.5, 9.0)	<0.001
Post-warning change in level, controls [b]	1.5 (0.8, 2.3)	<0.001
Post-warning change in trend, controls [c]	0.1 (0.004, 0.1)	0.031
Difference in pre-warning level (montelukast – controls)	155.6 (149.8, 161.4)	<0.001
Difference in pre-warning trend (montelukast – controls)	0.1 (-0.2, 0.4)	0.591
Difference in change in post-warning level (montelukast – controls)	0.3 (-1.1, 7.8)	0.144
Difference in change in post-warning trend (montelukast – controls)	-0.9 (-1.2, -0.6)	<0.001
Post-warning trend, montelukast [d]	-0.7 (-0.8, -0.6)	<0.001
Post-warning trend, controls [d]	0.1 (0.07, 0.2)	<0.001
Difference in post-warning trend (montelukast – controls)	-0.8 (-0.9, -0.7)	<0.001

[a] Control group of users of theophylline, biologics, zafirlukast and zileuton

[b] Level changes reflect the predicted change in prescription rates per 1000 between the last month of the pre-warning period (March 2020) and the first month of the post-warning period (April 2020).
[c] Trend changes reflect the predicted change in the slope of prescription rates per 1000 between the pre-warning period (October 2017 - March 2020, inclusive) and the post-warning period (April 2020 – December 2022).
[d] Estimates of the post-intervention linear trend were obtained using linear combinations of the pre-intervention trend and the change in trend.

© 2026 Shanmugam H et al. *JAMA Network Open*.

Supplemental Table 3. Multiple-group ITSA of changes in Montelukast prevalence after boxed warning relative to controls, excluding zafirlukast and zileuton [a]

	**Model estimate, per 1000 asthma patients per month (95% CI)**	***P* value**
Pre-warning baseline level	6.5 (5.9, 7.1)	<0.001
Post-warning change in level, controls [b]	1.1 (0.5, 1.8)	<0.001
Post-warning change in trend, controls [c]	0.05 (0.02, 0.1)	0.002
Difference in pre-warning level (montelukast – controls)	157.3 (151.6, 163.1)	<0.001
Difference in pre-warning trend (montelukast – controls)	0.1 (-0.2, 0.4)	0.591
Difference in change in post-warning level (montelukast – controls)	0.4 (-0.8, 8.1)	0.105
Difference in change in post-warning trend (montelukast – controls)	-0.9 (-1.2, -0.6)	<0.001
Post-warning trend, montelukast [d]	-0.7 (-0.8, -0.6)	<0.001
Post-warning trend, controls [d]	0.2 (0.01, 0.2)	<0.001
Difference in post-warning trend (montelukast – controls)	-0.8 (-0.9, -0.7)	<0.001

[a] Control group of users of theophylline, biologics

[b] Level changes reflect the predicted change in prescription rates per 1000 between the last month of the pre-warning period (March 2020) and the first month of the post-warning period (April 2020).
[c] Trend changes reflect the predicted change in the slope of prescription rates per 1000 between the pre-warning period (October 2017 - March 2020, inclusive) and the post-warning period (April 2020 – December 2022).
[d] Estimates of the post-intervention linear trend were obtained using linear combinations of the pre-intervention trend and the change in trend.

© 2026 Shanmugam H et al. *JAMA Network Open*.

Supplemental Table 4. Interrupted time series analysis estimates of changes in Montelukast use after March 2020 boxed warning announcement, with washout period [a]

	**Montelukast Prevalence, per 1000 asthma patients per month (95% CI)**	***P* value**	**Montelukast incidence, per 1000 non-user asthma patients per month (95% CI)**	***P* value**
Baseline level	160.7 (160.0, 165.5)	<0.001	5.9 (5.5, 6.2)	<0.001
Baseline trend	0.1 (-0.1, 0.04)	0.324	0.01 (-0.008, 0.03)	0.217
Post-warning change in level [b]	3.3 (-1.1, 7.7)	0.141	-1.7 (-2.2,-1.2)	<0.001
Post-warning change in trend [c]	-0.8 (-1.1, -0.5)	<0.001	-0.008 (-0.03, 0.01)	0.528
Post-warning trend [d]	-0.6 (-0.7, -0.5)	<0.001	0.005 (-0.01, 0.02)	0.508

[a] All analyses adjusted for seasonality and first-order autocorrelation. Washout period excluded March 2020 and April 2020.

[b] Level changes reflect the predicted change in prescription rates per 1000 between the last month of the pre-washout, pre-warning period (February 2020) and the first month of the post-washout, post-warning period (May 2020).

[c] Trend changes reflect the predicted change in the slope of prescription rates per 1000 between the pre-washout, pre-warning period (October 2017 - February 2020, inclusive) and the post-washout, post-warning period (May 2020 – December 2022).

[d] Estimates of the post-intervention linear trend were obtained using linear combinations of the pre-intervention trend and the change in trend.

© 2026 Shanmugam H et al. *JAMA Network Open*.
