# Supplementary material for: Changes in the Use of Montelukast for Asthma After a US Food and Drug Administration Boxed Warning
Source: JAMA Netw Open. 2026 May 22;9(5):e2614274. doi: 10.1001/jamanetworkopen.2026.14274 (PMC13197876; doi:10.1001/jamanetworkopen.2026.14274)
Supplement: Supplement 2. — Data Sharing Statement [file jamanetwopen-e2614274-s002.pdf]

Data Sharing Statement

Shanmugam. Changes in the Use of Montelukast for Asthma After a US Food and Drug Administration Boxed Warning. *JAMA Netw Open*. Published May 22, 2026. doi:10.1001/jamanetworkopen.2026.14274

Data
Data available: No

Additional Information
Explanation for why data not available: MarketScan data were accessed under a data use agreement with Merative; the company can be contacted directly by interested parties to license the data at their own expense.
